# Active treatment vs. expectant management of patent ductus arteriosus in preterm infants: critical analysis of a meta-analysis

**DOI:** 10.1186/s13052-026-02244-3

**Published:** 2026-04-10

**Authors:** Carlo Dani, Massimo Agosti, Daniele de Luca

**Affiliations:** 1 Department of Neurosciences, Psychology, Drug Research and Child Health, University of Florence, Florence, Italy; 2 Division of Neonatology, Careggi University Hospital of Florence, Largo Brambilla 3, 50134 Florence, Italy; 3 Department of Medicine and Technical Innovation, Pediatrics, Hospital “F. Del Ponte”, University of Insubria, Varese, Italy; 4 Division of Pediatrics and Neonatal Critical Care, A. Béclère Medical Center, Paris Saclay University Hospitals, Paris, France

## Abstract

A recent meta-analysis on the active versus expectant management of patent ductus arteriosus in preterm infants, including 10 randomized control trials involving 2035 infants, found that the incidence of death at 36 weeks or moderate to severe bronchopulmonary dysplasia (BPD) and of death was higher in the active treatment group. The accompanying editorial suggested that “*the use of early routine pharmacologic treatment no longer appears to be justifiable”.* However, this meta-analysis is based on highly heterogeneous studies due to different diagnostic criteria used to define hemodynamically significant PDA, postnatal age at which the echocardiography was performed, pharmacological treatment, high frequency of open label treatment in the placebo/expectant management group. Moreover, authors did not explain the reduction in mortality that they reported. We concluded that clinical and methodological heterogeneity among studies may not be detected by statistical heterogeneity tests, reducing the interpretability of the results and weakening causal inferences. The high occurrence of open-label treatment indicates the need to perform a per-protocol analysis, which would have been possible if the authors had performed an individual participant data network meta-analysis. Furthermore, the current lack of pathophysiological plausibility suggests caution in interpreting the results of this meta-analysis.

## Background

Buvaneswarran et al. have recently published a meta-analysis on the active versus expectant management of patent ductus arteriosus in preterm infants [1]. This meta-analysis included 10 randomized control trials involving 2035 infants [1]. They found that the incidence of death at 36 weeks or moderate to severe bronchopulmonary dysplasia (BPD) and of death was higher in the active treatment group vs. the expectant management group [1]. The study has been accompanied by an editorial in which it was suggested that ”*the adverse effects highlighted in the meta-analysis by Buvaneswarran et al. shift the balance of risks and benefits when considering how to manage an infant with a presymptomatic moderate or large PDA shunt during the first weeks. Previously*,* one might have considered early treatment (for its short-term benefits) to be equivalent to expectant management*,* since long-term morbidities appeared to be similar between the approaches. Now*,* with an increased risk of death*,* the use of early routine pharmacologic treatment no longer appears to be justifiable.*” [2]. The Editorial reported also that “*The concerns raised in the meta-analysis by Buvaneswarran et al. should not imply that the PDA shunt does not have a negative impact on the infant.***”**, and that ***“****Whether current ongoing and future RCTs*,* using improved selection criteria* *and more effective PDA closure strategies*,* will be able to find an at-risk group where early routine PDA closure might be able to decrease the incidence of BPD or other morbidities remains to be seen.”.* The Editorial does not comment on the methodology of the meta-analysis and the reliability of its results [2].

## Discussion

The conclusion of this meta-analysis seems too directive and not sufficiently justified, as we face an oversimplification of a phenomenon, the patent ductus arteriosus (PDA), which has a very complex pathophysiology and that the meta-analysis does not resolve due to many potential critical points.

First, the enrollment criteria, and in particular the echocardiographic criteria that were used in the different studies. (Table 1) It is evident that they are very different from each other, and in particular the RCTs of Hundscheid et al. [3] and de Waal [4] used only the criterion of PDA diameter > 1.5 mm, which is notoriously insufficient in itself to define a hemodynamically significant PDA (hsPDA) [5]. The result is that most likely in these studies, which enrolled 345 newborns, many patients were treated without requesting it because they did not have an hsPDA [3, 4] contradicting the editorial stating that “*since 2010*,* …treatment RCTs have made a concerted effort to include only those infants … at risk for PDA-related morbidities*” [2]. Furthermore, it is well known that even using composite echocardiographic criteria, the diagnosis of hemodynamically significant PDA is not coincident and reproducible [6]. This is even more true if echocardiography is performed at different times and, in fact, the meta-analyzed studies realized the scan in the first 12 h of life [7, 8], or between 24 and 72 h of life [3, 4, 9, 10], or in the second week of life [11–12]. (Table 1) Consistently, among the many details reported by the authors, these different diagnostic criteria were not reported and were given little consideration despite being fundamental for evaluating the homogeneity of the studies. Second, it should be added that several studies also enrolled newborns of 28–29 weeks of gestational age [4, 8–10], and others of 30 [13] and 31 [12, 14] rather than preterm infants with gestational age less than 28 weeks of gestation as suggested in the editorial [2]. These different characteristics indicate that the studied populations had PDA with different pathophysiology and different hemodynamic consequences. Mixing patients with different pathophysiology may be intriguing to have larger population but eventually reduces the accuracy and usefulness of findings. In other fields of critical care medicine a more explanatory rather than pragmatic approach is being preferred: this seems forgotten and absolutely needed for PDA too.

Second, in many analyzed studies, the percentage of infants in the placebo/conservative treatment group who received open label pharmacological treatment is very high, ranging from 0.4 [12] to 62.3% [7], and resulting in a total of 29.2% (297/1017). (Table 1) Therefore, one third of the patients in the placebo/conservative treatment group received pharmacological therapy but, according to the intention to treat principle, they were analyzed as if they had not done it. This alone would be enough to not be sure of the results of the meta-analysis, but even this aspect is neither detailed nor considered, although 7 out of 10 studies are reported by the authors in supplemental material as having bias due to deviations from intended intervention. In fact, when the authors wrote that patients in the placebo/conservative treatment group had a lower mortality or a lower frequency of periventricular leukomalacia, they should have considered that one in three newborns in this group received pharmacological treatment, which may have reduced mortality and narrowed the difference with the pharmacological control group. This is why the intention-to-treat analysis should be accompanied in such cases by a per-protocol analysis, which would have been possible if the authors had performed an individual participant data network meta-analysis (IPD-Mas). In fact, Individual Patient Data Meta-Analyses (IPD-MAs) are generally considered superior to Aggregate Data Meta-Analyses (AD-MAs) because they provide greater data standardization, enable more reliable subgroup analyses, reduce bias, and allow for more complex and precise investigations of treatment effects. For these reasons, IPD-MAs are often regarded as the “gold standard” of evidence synthesis, although they are considerably more resource-intensive and time-consuming to conduct. This view is widely supported in the literature [15]. 

**Table 1:** Main characteristics of studies included in the meta-analysis by Buvaneswarran et al. [1]

Reference and number of patients	Echocardiographic criteria for enrollment	Age at enrollment	Gestational age and birth weight criteria	Open label treatment in conservative/ placebo group and used drugs
Gupta et al. [8 ] (*n* = 422)	PDA diameter of ≥ 1.5 mm with pulsatile flow	< 72 h	23–28 wks	29.8% (*n* = 97) Ibuprofen
Hundscheid et al. [3 ] (*n* = 273)	PDA diameter > 1.5 mm and L-R shunt	24–72 h	< 28 wks	0.4 (*n* = 1) Ibuprofen
Roze et al. [6] *N* = 228	PDA diameter > 2.26 mm – (0.078 postnatal age in hours)	6–12 h < 28	< 28 wks	62.3% (*n* = 71) Ibuprofen
El-Khuffash et al. [9] (*n* = 60)	PDA risk score > 5.0 (calculated based on the following formula: (Gestation in weeks 1.304) + (PDA diameter in mm 0.781) + (Left ventricular output in mL/kg/min 0.008) + (maximum PDA velocity in m/s in cm/s 1.065) + (left ventricular a’ wave 0.470) + 41, where 41 is the constant of the formula)	36–48 h	< 29 wks	12% (*n* = 4) Ibuprofen
de Waal et al. [4] (*n* = 72)	PDA diameter > 1.5 mm	< 72 h - <29 d	< 29 wks	8% (*n* = 3) Ibuprofen or indomethacin
Clyman et al. [10] (*n* = 202)	Ductus diameter ≥ 1.5 mm (or a PDA: left pulmonary artery diameter ≥ 0.5) and 1 or more of the following echocardiographic criteria: (1) left atrium-to aortic root ratio ≥ 1.6, (2) ductus flow velocity ≤ 2.5 m/second or mean pressure gradient across the ductus ≤ 8 mmHg, (3) left pulmonary artery diastolic flow velocity > 0.2 m/second, and/or (5) reversed diastolic flow in the descending aorta.	6–14 gg	< 28 wks	48% (*n* = 47) Ibuprofen or paracetamol or indomethacin
Potsiurko et al. [13 ] (*n* = 208)	Unknown	< 72 h	< 32 wks and < 1500 g	Ibuprofen or paracetamol
Sosenko et al. [11 ] (*n* = 105)	Infants with HS PDA at enrollment were excluded from the study. Eligible: early, mild symptoms of a PDA (metabolic acidosis, mean blood pressure < weeks in gestation or requiring vasopressor support, bounding pulses/ hyperactive precordium, or systolic murmur). When one or more of the aforementioned symptoms was noted, an echocardiogram was performed. When the initial echocardiogram results were positive for PDA (defined as the presence of a PDA with either predominantly left-to-right or bidirectional shunt), infants were enrolled,	> 24 h - <14 days	23–32 wks and 500–1250 g	51% (*n* = 26) Ibuprofen
Sung et al. [12 ] (*n* = 142)	PDA diameter > 1.5 mm and L-R shunt	6 days	23–30 wks	0% Oral ibuprofen
Kluckow et al. [7 ] (*n* = 192)	Infants classified as having a large PDA were block randomized. The PDA diameters used were > 1.8 mm at postnatal age 3–5 h, > 1.6 mm at postnatal age 6–8 h and > 1.3 mm at postnatal age 9–12 h.	< 12 h	< 29 wks	40% (*n* = 19) Indomethacin

Third, the explanations for the better results observed in the expectation management group also seem unconvincing. When the authors state that “*pulmonary blood flow (PBF) was substantially lower in patients with more severe lung disease*” [1], they implicitly stated that pulmonary vascular resistance was increased, or not decreased, in these patients. How, then, could what they state in the next sentence occur: “*as lung disease worsened*,* PDA increased PBF*” [1]? In fact, increased pulmonary resistance in infants with more severe lung disease cannot physiologically coincide with increased left-to-right shunt. As for the hypothesized improvement in left ventricular cardiac output [1], it is not clear what it should derive from. Finally, if the authors [1] hypothesized an increase in pulmonary flow due to a left-to-right shunt through the PDA, this should cause a reduction in cerebral flow since the PDA steals it from the systemic circulation, contributing to increasing the risk of cerebral ischemia rather than reducing it. Moreover, the authors state that “*ductal patency resulted in higher systemic blood flow than when the ductus was ligated*,” [1] but it is well known that a hemodynamically significant PDA can reduce aortic, mesenteric, and cerebral blood flow and that the reduction in left ventricular output observed in post-ligation cardiac syndrome is caused by other, not fully understood, mechanisms [14]. On the other hand, the authors themselves are unable to explain the reduction in mortality that they reported and, wrote “*Based on our findings*,* it was not possible to explain the cause of the higher mortality rates seen in the active treatment group*.” [1]. This is the most critical point since a meta-analysis should not be a mere mathematical exercise but must include an appraisal of pathobiological and pathophysiological plausibility. In absence of this, results of meta-analysis are unsupported and can be explained by statistical heterogeneity and other phenomena. For example, amongst these phenomena, we noted that there was a slight preponderance of sepsis which can have caused the higher mortality in the active treatment group. How can the increase in sepsis be explained? Sepsis can hardly be caused by the PDA pharmacological treatment. Without pathophysiological plausibility, statistically significant associations are more likely to represent artefacts rather than true causal relationships. The lack of an elucidated pathophysiological mechanism does not automatically imply that the result is incorrect, but it weakens the reliability of the meta-analysis and justifies a cautious interpretation of its results.

This is why plausibility analysis and reporting are recommended in every meta-analysis by many authors, such as Dailey et al. [16]. On the other hand, if the mechanism that may explain the adverse effects of pharmacological closure of hsPDA is unknown, and most of the studies analyzed concern ibuprofen, it is not possible to draw the conclusion that any treatment of hsPDA, including that with paracetamol or indomethacin or transcatheter closure, can have the same effects.

Fourth, clinical heterogeneity should not be confused with the statistical heterogeneity of the studies that the authors refer to when they write “*There was also minimal study heterogeneity*,* suggesting insignificant variation in the true treatment effect size*.“ [1]. Indeed, to assess heterogeneity, they specified values of τ^2^ = 0, I^2^ = 0%, and H^2^ = 1.00 for both death and the composite of death or moderate to severe BPD [1]. However, the used heterogeneity test can detect only differences in the final numerical results (statistical heterogeneity), not in the clinical or methodological characteristics of the studies (clinical heterogeneity). Although statistical heterogeneity was low, the included studies differed substantially in terms of gestational age, postnatal age, diagnostic criteria, and pharmacological interventions, indicating the presence of clinical and methodological heterogeneity that may not be captured by statistical measures alone. Melsen et al. effectively explained that with high levels of clinical heterogeneity, even meta-analyses with low statistical heterogeneity have low predictive values [17]. Furthermore, no individually meta-analyzed study has demonstrated an increased risk of mortality due to drug treatment of PDA, and therefore, the interpretation of this combined detrimental effect in highly heterogeneous studies becomes problematic as it may not reflect a causal drug-related effect but the effect of clinical confounders.

On the other hand, one cannot expect that the summation of studies with methodological limitations can eliminate these limitations by resolving them.

## Conclusions

In conclusion, this meta-analysis provides results that must be interpreted with great caution because it is based on highly heterogeneous studies in which 29.2% of infants in the placebo/conservative group received open-label pharmacological treatment and, consistently, does not support its results with pathophysiological plausible mechanisms. Although statistical heterogeneity was low, the included studies differed substantially in terms of gestational age, postnatal age, diagnostic criteria, and pharmacological interventions, indicating the presence of clinical and methodological heterogeneity that may not be captured by statistical measures alone. The high occurrence of open-label treatment indicates the need to perform a per-protocol analysis, which would have been possible if the authors had performed an IPD-MAs. Furthermore, the lack of an elucidated pathophysiological weakens the reliability of the meta-analysis and justifies a cautious interpretation of its results.

## Data Availability

Not applicable.

## Abbreviations

BPD: Bronchopulmonary dysplasia
PDA: Patent ductus arteriosus
PBF: Pulmonary blood flow
